# Socioeconomic Barriers for Newly Transferred Young Adults With Congenital Heart Disease

**DOI:** 10.1016/j.cjcpc.2025.08.005

**Published:** 2025-09-01

**Authors:** Jodie Beuth, Sara Thorne, Brigitte Mueller, David Barron, Adrienne Kovacs, Rafael Alonso-Gonzalez, Heather Ross, Erwin Oechslin, Loretta TS. Ho, Jane Heggie

**Affiliations:** a Department of Anaesthesia and Perfusion Services, the Prince Charles Hospital, Brisbane, Queensland, Australia; b Division of Cardiology, Peter Munk Cardiac Centre, Toronto General Hospital, Toronto, Ontario, Canada; c Ted Rogers Computational Program, Peter Munk Cardiac Centre, Toronto General Hospital, Toronto, Ontario, Canada; d Division of Cardiac Surgery, Peter Munk Cardiac Centre, Toronto General Hospital, Toronto, Ontario, Canada; e Division of Cardiac Surgery, Hospital for Sick Children, Toronto, Ontario, Canada; f Equilibria Psychological Health, Toronto, Ontario, Canada; g Division of Cardiology, Toronto Congenital Cardiac Centre for Adults, Peter Munk Cardiac Centre, Toronto General Hospital, Toronto, Ontario, Canada; h Ted Rogers Centre for Heart Research, University of Toronto, Toronto, Ontario, Canada; i Department of Anesthesia and Pain Medicine, University of Toronto, Toronto General Hospital, Toronto, Ontario, Canada

**Keywords:** socioeconomic barriers, adult congenital heart disease, transfer of care

## Abstract

**Background:**

This study aimed to ascertain socioeconomic factors affecting successful transfer to adult congenital cardiology and cardiac surgical services in Ontario.

**Methods:**

Patients with congenital heart disease (CHD) referred from a pediatric CHD program to an adult CHD (ACHD) center between January 1, 2004, and December 31, 2015, were identified. The prevalence of (1) failed transfer (FT), (2) lost to follow-up (LTFU), and (3) cardiac surgery (CS) during the extended study period of 2004-2018 was investigated. Socioeconomic variables associated with FT, LTFU, and CS were explored using Environics data associated with postal code at the time of transfer.

**Results:**

A total of 2196 patients were referred from the pediatric to ACHD center between 2004 and 2015. Within this cohort, 11% had FT and 25% had LTFU; there was a 2% overlap between the FT and LTFU groups. A total of 106 patients (4.8%) underwent CS. Age at referral (odds ratio [OR]: 0.591, *P* < 0.001) and being unable to travel to work by car (OR: 0.986, *P* < 0.001) were both associated with FT, though the latter is not clinically relevant. Residential addresses with lower income (OR: 0.976, *P* = 0.016) were associated with LTFU. Factors associated with CS were higher household income (*P* < 0.001), access to a car for travel to work (*P* < 0.001), Canadian citizenship (*P* = 0.041), and French or English as the primary language in the home (*P* = 0.038).

**Conclusions:**

Socioeconomic factors are associated with access to specialized ACHD services among young adults. Strategies to ensure equity in care should be explored.

## Résumé

**Contexte:**

Cette étude visait à déterminer les facteurs socio-économiques influençant la réussite du transfert des patients vers les services de cardiologie congénitale et de chirurgie cardiaque pour adultes en Ontario.

**Méthodologie:**

Nous avons identifié des patients atteints d’une cardiopathie congénitale suivis dans le cadre d’un programme pédiatrique de prise en charge des cardiopathies congénitales et orientés vers un centre de traitement des cardiopathies congénitales pour adultes entre le 1^er^ janvier 2004 et le 31 décembre 2015. Nous avons évalué la prévalence (1) de l’échec de l’orientation, (2) de la perte de vue au suivi et (3) de la chirurgie cardiaque pendant l’étude et sa phase de prolongation, soit de 2004 à 2018. Nous avons étudié les variables socio-économiques associées à l’échec de l’orientation, à la perte de vue au suivi et à la chirurgie cardiaque à l’aide des données Environics associées au code postal au moment de l’orientation.

**Résultats:**

Au total, 2 196 patients ont été orientés d’un centre pédiatrique vers un centre pour adultes entre 2004 et 2015. Dans cette cohorte, 11 % ont connu un échec d’orientation et 25 % ont été perdus de vue au suivi. Un chevauchement de 2 % a été observé entre les deux groupes. Au total, 106 patients (4,8 %) se sont prêtés à une chirurgie cardiaque. L’âge au moment de l’orientation (rapport de cotes [RC] : 0,591; *p* < 0,001) et l’incapacité de se rendre au travail en voiture (RC : 0,986; *p* < 0,001) ont été tous deux associés à l’échec de l’orientation, même si ce dernier critère n’est pas pertinent sur le plan clinique. Les lieux de résidence associés à un faible revenu (RC : 0,976; *p* = 0,016) ont été associés à la perte de vue au suivi. Les facteurs associés à la chirurgie cardiaque sont un revenu ménager plus élevé (*p* < 0,001), l’accès à une voiture pour se rendre au travail (*p* < 0,001), la citoyenneté canadienne (*p* = 0,041) et le français ou l’anglais comme langue principale à la maison (*p* = 0,038).

**Conclusions:**

Les facteurs socio-économiques sont associés à l’accès aux services spécialisés de prise en charge des cardiopathies congénitales chez les jeunes adultes. Il convient d’explorer les stratégies possibles pour garantir l’équité dans les soins.

Factors affecting young adults’ transfer and continued access to specialist adult congenital heart disease (ACHD) services and cardiac surgical services are not well understood.

Serial prevalence studies in the province of Québec have demonstrated that the median survival of patients living with severe forms of congenital heart disease (CHD) has risen from 11 years in 1985, 17 years in 2000, to 25 years in 2010.^1^^,^^2^ The ratio of adults (>18 years) with CHD to children (<18 years) surpassed 2:1 in 2010.^1^^,^^2^ A recent Swedish study of >37,000 patients with CHD who reached the age of 18 years and were born between 1950 and 1999 found that 75% of patients with CHD alive at 18 years of age lived past middle age and became sexagenarians, with a notable mortality risk decline in those patients born after 1975.^3^

Successful transfer and continuity of care are important with increasing survivorship into the adult age. A 2004 cross-sectional cohort study of 360 patients, aged 19-21 years, for whom practice guidelines indicated that they should undergo annual ACHD review, was conducted in Ontario.^4^ Of these patients, only 47% successfully transferred, with 22% of the cohort not being seen after the age of 10 years.^4^ A self-reporting questionnaire of the cohort revealed that 27% of patients had not attended a CHD appointment because they had reached 18 years of age.^4^ Subsequently, substantial efforts have been made to improve access to care for the ACHD population, with publication of a 2019 national consensus defining transition with strategies to improve successful transfer across Canada.^5^

The present study aimed to ascertain socioeconomic factors in the patients’ environment that may affect access to ACHD care and access to cardiac surgical services in Ontario. Specifically, we investigated the prevalence and associated sociodemographic variables with.

(1) ACHD clinic attendance within 3 years of transfer from pediatric center,
(2) ACHD clinic attendance without a gap in care of 5 years or more, and
(3) cardiac surgery (CS) among transferred patients.

## Methods

After Québec, Ontario is Canada’s second largest province with a population just over 15 million people in a land area larger than France and Spain combined. In accordance with the Canada Health Act, all CHD services are accessed through a publicly funded health care system with universal health coverage. There is no private health care system.

### Study design

This was a retrospective study using deidentified data; approval by the Research Ethics Board (REB) did not require patient consent. The REB approved examination of the administrative transfer process of patients with CHD having reached 18 years of age who were formally referred as new patients to the ACHD clinic, including use of 6-digit postal codes. The referral site was the Hospital for Sick Children (HSC) with transfer of adult care to the adjacent University Health Network (UHN) site, Toronto General Hospital (TGH). Referred patients were administratively assigned a UHN identifier (medical record number), and HSC clinical records were electronically transferred into the patients’ UHN chart. These charts were reviewed for referral letter, address at the time of transfer (defined by a 6-digit postal code), and examined for (1) successful transfer and participating in medical care without a gap exceeding 5 years, (2) successful transfer (appointment attendance within 3 years of transfer), and (3) gaps in care 5 years or more. The cohort was then matched to an existing cardiac surgical database for the period 2004-2018, allowing for the patients referred in 2015 to successfully transfer.

Socioeconomic and demographic data were obtained from Environics Analytics, a Canadian analytics and marketing company that specializes in geodemographic segmentation and modeling based on anonymized data aggregated to the 6-digit postal code level. Environics compiles data from multiple sources including the Canadian Census, current economic indicators, postcensal estimates from federal and provincial governments, household surveys, and immigration statistics. These data are used to generate neighborhood-level indicators such as income, educational attainment, unemployment, language proficiency, and access to transportation. Environics data products have been widely used in applied research and policy analysis. The REB permitted sharing of patient deidentified postal codes with Environics Analytics.

To geocode patient addresses, we used Service Objects (https://www.serviceobjects.com/, accessed and downloaded on February 4, 2022), a service providing latitude and longitude coordinates corresponding to the geographic center of Canadian postal code areas. These coordinates were used to link patients' residential postal codes to neighborhood-level sociodemographic data from Environics.

Data on travel to work by car are collected in the Canadian Census and made available through Environics Analytics. Similarly, unemployment levels are based on Statistics Canada data modeled at the postal code level and reflect the proportion of individuals actively seeking but unable to obtain work.

The research reported in this paper adhered to STROBE (Strengthening the Reporting of Observational studies in Epidemiology) guidelines for reporting observational studies.^6^

### Participants

The study population was a cohort of patients with CHD who turned 18 years of age, 2004-2015 inclusive, with at least 3 years of follow-up through to 2018, and who were transferred from HSC to the tertiary ACHD program at TGH.

### Outcome measures

Four outcomes were defined:

(1) Successful transfer: An ACHD clinic visit within 3 years of referral from a pediatric center plus retention in ACHD care (without a gap of ≥5 years).
(2) Failed transfer (FT): nonattendance at an ACHD clinic visit within 3 years of referral.
(3) Loss to follow-up (LTFU): nonattendance at an ACHD clinic visit ≥5 years after transfer during the study follow-up period. (FT and LTFU were not mutually exclusive.)
(4) CS during the extended study period of 2004-2018.

### Statistical analyses

Data were first described using summary statistics. Comparisons between the groups were evaluated using Wilcoxon rank-sum tests for continuous and Fisher exact tests for dichotomous and polytomous variables. Continuous variables were summarized using medians and interquartile ranges. Categorical variables were summarized in terms of the numbers and proportions of patients.

Before model fitting, we removed 157 patients with more than 80% of data missing. The remaining missing data (one value for age, work from home by employed, unemployment rate, or travel to work by car) were imputed using the median.

To identify predictors of LTFU and FT, we fitted a least absolute shrinkage and selection operator (LASSO) logistic regression model using 10-fold cross-validation via the cv.glmnet function in the glmnet R package. LASSO shrinks some regression coefficients to zero, thereby performing variable selection. This approach was chosen because of the presence of multicollinearity among several predictors (eg, educational variables), as it helps stabilize coefficient estimates and improve model interpretability. A final logistic regression model with the selected covariates was run to obtain odds ratios (ORs) with 95% confidence intervals (95% CIs) and *P* values.

## Results

### Participants

Hospital records identified 2196 patients referred from HSC to TGH between 2004 and 2015, who were included in the study.

The socioeconomic characteristics of the study sample are presented in Table 1. Of the 2196 patients initially identified, 70 did not have a valid postal code and 9 resided outside of Ontario at the time of data abstraction.

**Table 1:** Participant socioeconomic characteristics (n = 2196)

Variables	N∗	Statistic†
Age at referral (y)	2184	17 (17-18)
Median household income (constant year 2015 $)	2075	85,899 (58,416-118,251)
Education (% of population 15 years or over)		
No certificate, diploma, or degree	2008	14 (11-19)
High school certificate or equivalent	2008	28 (24-32)
Apprenticeship/trade certificate or diploma	2008	5 (3-8)
College/nonuniversity certificate or diploma	2008	22 (16-26)
University certificate/diploma below bachelor	2008	1.3 (0-2.6)
University degree	2008	25 (16-38)
Bachelor degree	2008	19 (12-26)
Above bachelor degree	2008	7 (3-13)
Employment (% of population 15 years or over)		
Employed	2008	59 (51-67)
Unemployment rate	2007	9.4 (5.3-14.4)
Worked from home by employed	2007	20 (18-23)
Travel		
Distance to TGH (km)	2096	34 (18-78)
Distance to transport hub or TGH (km)	2096	32 (17-68)
Travel to work by car (% of all forms of travel)	2007	91 (76-97)
Travel to work by car as driver (% of all forms of travel)	2007	88 (66-95)
Citizenship and immigration (in % of population)		
Visible minority total	2008	25 (6-60)
Total immigrant	2008	28 (12-50)
Noncitizen	2008	6 (2-11)
First generation	2008	30 (13-523)
Second generation	2008	25 (18-31)
Third generation or more	2008	42 (16-67)
Neither French nor English speaking	2008	1.2 (0-4)
Regions		
Rural	2126	253 (11.9)
Referral address	2136	
Greater Toronto Area		1674 (78.4)
Rest of Ontario		453 (21.2)
Rest of Canada		9 (0.4)

TGH, Toronto General Hospital.

∗ Number of available data.

† Statistic: median (25th percentile to 75th percentile) for continuous variables and counts (percentages) for all other variables.

### Transfer and retention outcomes

For patients with successful transfers, the median time between transfer and the first ACHD clinic visit was 15 months (interquartile range: 10-26 months). Of 2196 patients, FT occurred in 245 (11%) and LTFU occurred in 549 (26%) (Table 2). There was a 2% overlap between FT and LTFU, meaning 31 patients FT and presented after a gap in care of over 5 years. Of 9 interprovincial patients, 5 were successfully transferred, 1 patient FT, and data were unavailable for 2 patients from Newfoundland and 1 patient from Saskatchewan (Table 3).

**Table 2:** Outcome of transfer

Outcome of transfer	N	Statistic
Transfer time (mo), median (interquartile range)	1844	15 (10-26)
Failure to transfer (gap in care >3 years), n (%)	1844	245 (11)
Lost to follow-up (gap in care >5 years), n (%)	2130	549 (26)

**Table 3:** Cohort description stratified by failure to transfer

Variable	Successful transfer		Failed transfer		*P* value
	N∗	Statistic†	N∗	Statistic†	
Age at referral (y)	1599	17 (17-18)	244	17 (17-18)	<0.001
Distance to TGH (km)	1561	35.56 (20-80)	242	28.41 (13-66)	0.002
Distance to transport hub or TGH (km)	1561	32.37 (18-702)	242	27.55 (13-64)	0.013
No certificate/diploma/degree‡	1495	14.10 (10-19)	233	15.38 (11-19)	0.085
Travel to work by car§	1494	92.07 (78-97)	233	88.64 (67-96)	<0.001
Travel to work by car as driver§	1494	88.94 (69-95)	233	83.47 (49-94)	<0.001
Referral address	1594		245		0.07
Greater Toronto Area		1228 (77)		204 (83.3)	
Rest of Ontario		361 (22.6)		40 (16.3)	
Rest of Canada		5 (0.3)		1 (0.4)	

TGH, Toronto General Hospital.

∗ Number of available data.

† Statistic: median (25th percentile to 75th percentile) for continuous variables and counts (percentages) for all other variables.

‡ Percentage of population above 15 years of age.

§ Percentage of all forms of travel to work.

In univariable logistic regression analysis, age at referral was significantly associated with FT (Tables 3 and 4). Specifically, for each additional year in age, the odds of FT decreased by approximately 40% (OR: 0.598; 95% CI: 0.501-0.714; *P* < 0.001). This association remained strong and statistically significant in the multivariable model (OR: 0.591; 95% CI: 0.493-0.707; *P* < 0.001).

**Table 4:** Univariable and multivariable odds ratios (ORs) and 95% confidence intervals (CIs) for failed transfer

Variable	Univariable	*P* value	Multivariable	*P* value
	OR (95% CI)		OR (95% CI)	
Age at referral	0.598 (0.501-0.714)	<0.001	0.591 (0.493-0.707)	<0.001
Median household income in $10,000	0.994 (0.974-1.014)	0.54	–	
Distance to transport hub or TGH	0.997 (0.995-1.000)	0.08	–	
Rural (ref: urban)	1.332 (0.895-1.981)	0.158	–	
No certificate, diploma or degree†	1.014 (0.996-1.032)	0.128	–	
High school certificate or equivalent†	0.997 (0.978-1.016)	0.75	–	
Apprenticeship or trades certificate or diploma†	0.985 (0.951-1.020)	0.39	–	
College, CEGEP or other nonuniversity certificate or diploma†	0.988 (0.968-1.007)	0.22	–	
University certificate or diploma below bachelor†	0.984 (0.930-1.042)	0.59	–	
University degree†	1.001 (0.993-1.010)	0.79	∗	
Bachelor's degree†	0.995 (0.981-1.009)	0.5	–	
Above bachelor's†	1.012 (0.995-1.029)	0.157	–	
Employed†	0.997 (0.987-1.008)	0.63	–	
Visible minority total‡	1.003 (0.998-1.007)	0.23	–	
Total immigrant‡	1.004 (0.998-1.011)	0.183	∗	
Worked from home by employed (%)	0.995 (0.972-1.019)	0.7	–	
Unemployment rate (%)	1.009 (0.992-1.026)	0.31	–	
Travel to work by car§	0.987 (0.981-0.993)	<0.001	0.986 (0.980-0.993)	<0.001
Travel to work by car as driver§	0.990 (0.985-0.995)	<0.001	∗	
Noncitizen‡	1.007 (0.989-1.024)	0.46	–	
First generation‡	1.004 (0.998-1.011)	0.165	∗	
Second generation‡	1.014 (0.998-1.030)	0.078	1.010 (0.991-1.030)	0.31
Third generation or more‡	0.996 (0.990-1.001)	0.086	∗	
Neither French nor English speaking‡	0.998 (0.963-1.034)	0.91	–	
Referral address rest of Ontario (ref: Greater Toronto Area)	0.642 (0.445-0.928)	0.018	0.753 (0.483-1.175)	0.21

Variables with an asterisk ∗ were excluded from consideration as potential risk factors due to high (>0.9) correlation with other variables. Variables with en dash (–) were not selected as risk factors by the LASSO regression.

CEGEP, collège d'enseignement général et professionnel; LASSO, least absolute shrinkage and selection operator; TGH, Toronto General Hospital.

† Percentage of population above 15 years of age.

‡ Percentage of population.

§ Percentage of all forms of travel to work.

Travel to work by car (as a percentage of all modes of commuting) was significantly associated with lower odds of FT in both univariable (OR: 0.987; 95% CI: 0.981-0.993; *P* < 0.001) and multivariable analyses (OR: 0.986; 95% CI: 0.980-0.993; *P* < 0.001). This is statistically significant because of the large sample size but not clinically significant.

Residence outside the Greater Toronto Area (GTA) was associated with significantly lower odds of FT in univariable analysis (OR: 0.642; 95% CI: 0.445-0.928; *P* = 0.018), but this association was no longer statistically significant after adjustment for other factors (OR: 0.753; 95% CI: 0.483-1.175; *P* = 0.21) (Table 4).

Patients who were LTFU were more likely to live in areas with lower average income, lower proportion of people who travel to work by car, higher unemployment levels, higher proportion of people without citizenship, nonofficial language use (neither French nor English), and lower educational attainment (ie, no certificate, diploma, or degree) compared with those who were not LTFU (Table 5). Patients with FT and LFTU were more often from neighborhoods, with a higher percentage of people without certificate or diploma (*P* = 0.041) (Tables 6 and 7). Using LASSO as a variable selection method confirmed the relevance of some of these factors. However, only income remained significant in the final model, indicating that the effect of these socioeconomic and educational factors might be captured by income. Participants from areas with higher median household income were less likely to be LTFU. Specifically, for every $10,000 CAD increase in household income, the odds of LTFU decreased by approximately 2.4% after accounting for other factors (Table 8). All variables were derived from Environics’ modeled neighborhood-level sociodemographic profiles.

**Table 5:** Description of variables associated with lost to follow-up

Variable	Retention in care		Lost to follow-up		*P* value
	N∗	Statistic†	N∗	Statistic†	
Age at referral (y)	1579	17 (17-18)	549	17 (17-18)	0.28
Median household income (constant year 2015 CAD$)	1524	88,888 (61,103-121,120)	514	80,000 (52,441-110,835)	<0.001
Travel to work by car‡	1472	92 (778-97)	500	89 (70-96)	0.006
Noncitizen§	1473	5.56 (1.5-10.6)	500	6.67 (1.8-12.7)	0.011
Neither French nor English speaking§	1473	1.15 (0-3.7)	500	1.66 (0-4.0)	0.018
No certificate, diploma or degree‖	1473	14.16 (10-187)	500	14.94 (11-20.0)	0.022
Unemployment rate (%)	1472	8.97 (5.0-14.3)	500	9.85 (6.2-14.8)	0.005

∗ Number of available data.

† Statistic: median (25th percentile to 75th percentile) for continuous variables and counts (percentages) for all other variables.

‡ Percentage of all forms of travel to work.

§ Percentage of population.

‖ Percentage of population above 15 years of age.

**Table 6:** Univariable and multivariable odds ratios (ORs) and 95% confidence intervals (CIs) for lost to follow-up

Variable	Univariable	*P* value	Multivariable	*P* value
	OR (95% CI)		OR (95% CI)	
Age at referral	1.044 (0.987-1.103)	0.13	1.047 (0.990-1.107)	0.107
Median household income in $1000	0.965 (0.946-0.984)	<0.001	0.976 (0.956-0.995)	0.016
Distance to transport hub or TGH	1.000 (0.998-1.002)	0.88	–	
Rural (ref: urban)	1.058 (0.775-1.445)	0.72	–	
No certificate, diploma, or degree†	1.016 (1.003-1.029)	0.018	–	
High school certificate or equivalent†	1.013 (0.999-1.028)	0.071	–	
Apprenticeship or trades certificate or diploma†	1.008 (0.984-1.034)	0.52	–	
College, CEGEP or other nonuniversity certificate or diploma†	0.997 (0.983-1.012)	0.73	–	
University certificate or diploma below bachelor†	0.981 (0.940-1.024)	0.39	–	
University degree†	0.994 (0.987-1.000)	0.067	∗	
Bachelor's degree†	0.992 (0.982-1.003)	0.137	–	
Above bachelor's†	0.987 (0.973-1.001)	0.062	–	
Employed†	0.990 (0.982-0.998)	0.017	0.997 (0.987-1.006)	0.49
Visible minority total‡	1.003 (1.000-1.006)	0.079	–	
Total immigrant‡	1.002 (0.997-1.006)	0.53	∗	
Worked from home by employed (%)	0.989 (0.972-1.007)	0.23	–	
Unemployment rate (%)	1.017 (1.005-1.030)	0.007	1.006 (0.992-1.021)	0.39
Travel to work by car§	0.992 (0.987-0.997)	0.002	0.996 (0.991-1.002)	0.164
Travel to work by car as driver§	0.994 (0.990-0.998)	0.003	∗	
Noncitizen‡	1.019 (1.007-1.032)	0.003	1.010 (0.996-1.024)	0.168
First generation‡	1.002 (0.998-1.007)	0.33	∗	
Second generation‡	0.996 (0.984-1.007)	0.45	–	
Third generation or more‡	0.999 (0.995-1.003)	0.57	∗	
Neither French nor English speaking‡	1.018 (0.993-1.043)	0.158	–	
Referral address rest of Ontario (ref: Greater Toronto Area)	1.053 (0.825-1.344)	0.68	–	

Variables with an asterisk (∗) were excluded from consideration as potential risk factors due to high (>0.9) correlation with other variables. Variables with en dash (–) were not selected as risk factors by the LASSO regression.

CEGEP, collège d'enseignement général et professionnel; TGH, Toronto General Hospital; LASSO, least absolute shrinkage and selection operator.

† Percentage of population above 15 years of age.

‡ Percentage of population.

§ Percentage of all forms of travel to work.

**Table 7:** Summary table of successful transfer, failed transfer (FT), lost to follow-up (LTFU), and patients who had cardiac surgery (patients who underwent cardiac surgery were not mutually exclusive from the successful transition)

Variable	FT		LTFU		Successful transfer		Cardiac surgery		*P* value
	N∗	Statistic†	N∗	Statistic†	N∗	Statistic†	N∗	Statistic†	
Age at referral (y)	212	17 (17-18)	539	17 (17-18)	1325	17 (17-18)	106	17 (17-18)	<0.001
Median household income‡	209	83,111 (53,734-118,285)	505	80,000 (51,977-111,517)	1259	89,751 (62,667-121,154)	98	90,400 (63,094-123,237)	<0.001
Education									
No certificate, diploma, or degree§	201	15 (11-19)	491	15 (10-20)	1217	14 (10-19)	98	14 (10-19)	0.041
High school certificate or equivalent§	201	28 (23-32)	491	28 (23-32)	1217	28 (23-32)	98	27 (24-31)	0.60
College/CEGEP or other certificate or diploma§	201	21 (16-26)	491	21 (16-26)	1217	22 (16-26)	98	22 (18-26)	0.22
Apprenticeship, trade certificate or diploma§	201	4.1 (1.9-7.9)	491	5.1 (2.6-8.1)	1217	5 (2.8-8.1)	98	6.3 (3.4-8.3)	0.044
University degree§	201	27 (15-38)	491	25 (16-36)	1217	26 (17-38)	98	25 (17-36)	0.38
Bachelor degree§	201	19 (11-26)	491	18 (12-25)	1217	19 (12-26)	98	18 (13-25)	0.39
Above bachelor degree§	201	8 (4-15)	491	7 (3-12)	1217	7 (4-13)	98	8 (4-11)	0.26
Employment									
Unemployed (%)	201	9 (6-14)	491	9.8 (7-15)	1216	9.1 (5-14)	98	10 (6-15)	0.023
Travel									
Distance to TGH (km)	210	27 (13-66)	510	33 (16-79)	1272	35 (19-78)	100	42 (23-74)	0.005
Travel to work by car^‖^	201	89 (67-96)	491	88.91 (70-96)	1216	92 (79-97)	98	92 (80-97)	<0.001
Travel to work by car as driver‖	201	84 (49-93)	491	86 (57-94)	1216	89 (70-95)	98	89 (72-95)	<0.001
Citizenship and immigration									
Noncitizen (% of population)	201	5 (2-11)	491	7 (2-13)	1217	6 (1-11)	98	5 (1-10)	0.041
Neither French nor English speaking (% of population)	201	1.5 (0-4)	491	1.7 (0-4)	1217	1.1 (0-3.6)	98	0.7 (0-3.6)	0.046

CEGEP, collège d'enseignement général et professionnel; TGH, Toronto General Hospital.

∗ Number of available data.

† Statistic: median (25th percentile to 75th percentile) for continuous variables and counts (percentages) for all other variables.

‡ Median household income constant year 2015 CAD$.

§ Percentage of population above 15 years of age.

‖ Percentage of all forms of travel to work.

**Table 8:** Summary of 106 cardiac surgical patients and transfer status (stratified by having either a gap in care 5 years and/or a failed transfer vs successful transfer without any gap in care)

Cardiac surgical patients	Lost to follow-up/failed transfer		Successfully transferred		*P* value
Variable	N	Statistic	N	Statistic	
Age at referral (y), median (IQR)	10	18 (17-18)	95	17 (17-18)	0.03
Age at surgery (y), median (IQR)	10	24 (23-25)	95	22 (21-25)	0.22
ACHD category, n (%)	10		93		0.29
Single ventricle noncyanotic∗		0 (0)		5 (5)	
Cyanotic		0 (0)		6 (6)	
2 Ventricle subaortic RV		2 (20)		5 (5)	
2 Ventricle subaortic LV		8 (80)		77 (83)	
Previous surgery, n (%)	10		95		
0		0 (0)		12 (13)	
1		4 (40)		29 (31)	
2		4 (40)		29 (31)	
3+		2 (20)		25 (26)	
Meld IX score, median (IQR)	10	6 (6-10)	75	6 (6-9)	0.96

ACHD, adult congenital heart disease; IQR, interquartile range; LV, left ventricle; RV, right ventricle.

### Cardiac surgery

**Figure 1:**
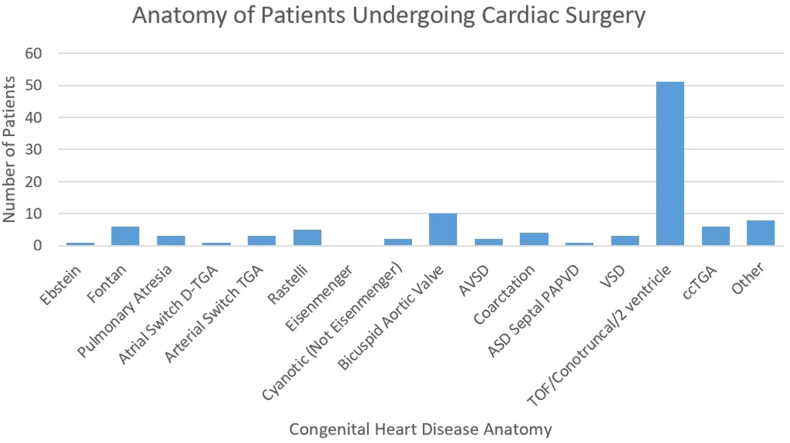
Summary of 106 cardiac surgical patients and anatomy. ASD, atrial septal defect; AVSD, atrioventricular septal defect; ccTGA, congenitally correction transposition of the great arteries; D-TGA, dextr-transposition of the great arteries; TOF, tetralogy of fallot; VSD, ventricular septal defect.

**Figure 2:**
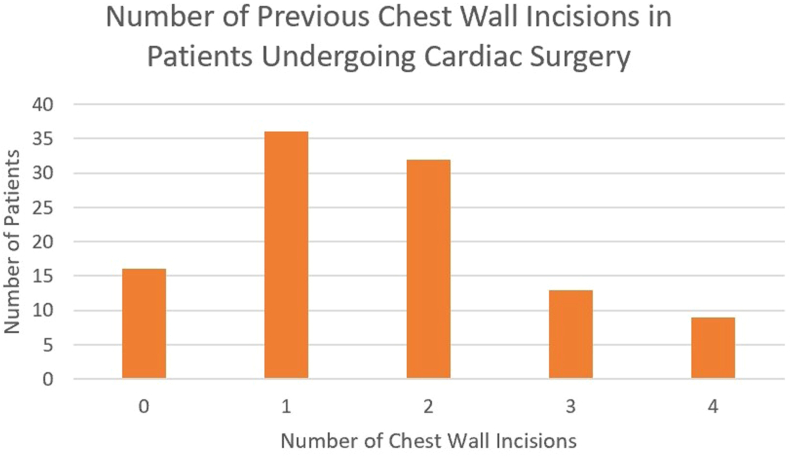
Summary of 106 cardiac surgical patients and previous chest wall incisions.

A total of 106 patients (4.8%) underwent CS in the observation period. Compared with patients who did not undergo surgery, those who did had postal codes associated with higher household income (*P* < 0.001) and a greater proportion of people who travel to work by car (*P* = 0.041) and spoke French or English (*P* = 0.038). Figures 1 and 2 outline congenital pathologies and previous chest wall incisions in those patients undergoing CS.

## Discussion

This study highlights factors such as income and transportation that can impact the ability of young people to navigate transfer to adult cardiac care services. Our study raises concerns that despite concerted efforts to improve the transfer process from pediatric to adult care via a targeted referral pathway with appropriate capacity, 11% of patients did not attend ACHD clinic appointments within 3 years of referral and 26% had a gap in care of ≥5 years after transfer. A small percentage of patients (2%) FT and presented more than 5 years after the transfer referral.

International guidelines recommend formal transition programs to improve rates of transfer and hence improve outcomes.^7^^,^^8^ Even in countries with universal public health care, there are significant proportions of the CHD population who are not referred along a formal process and are not managed into adult life by ACHD specialists.^9^ A 2007 study of 15 ACHD centers in Canada found that, of the estimated 96,000 patients with ACHD in Canada, only 21,879 were being regularly followed.^10^ A 2009 study reported significant lapses in care in Québec CHD patients younger than 18 years, even before initiation of the vulnerable phase of transfer to an ACHD facility.^11^ Older age was associated with successful transfer in our study, although our cohort was limited to 17- and 18-year-olds (Table 3). Although American guidelines recommend a flexible age of transfer, typically from 18 to 21 years, we are unaware of any dialogue within Ontario regarding consideration of a flexible age of transfer.^12^ A longitudinal study in Québec from 1997 to 2005 found an increase in the referral to specialized ACHD centers after the publication of the Canadian Consensus Guidelines and a reduction in mortality for those patients followed at a reference ACHD center.^13^^,^^14^

In Australia, the National Strategic Action Plan for Childhood Heart Disease 2019 highlighted the impact of deficiencies in infrastructure available to manage the growing ACHD population and the proportion of this population that resides in regional areas.^15^ This resulted in the first Australian National Standards of Care for Childhood-onset Heart Disease, which highlighted at-risk groups including the indigenous population, ethnic backgrounds, young patients aged 15-24 years transferring from pediatric to adult services, and those in rural and remote locations.^16^ Geographically, the Australian perspective may be comparable to that seen in this Canadian population, with over 28% of the population in Australia located in regional or remote areas requiring travel of more than 100 km to access an ACHD center.^16^ Because of this geographical isolation, a large proportion of the ACHD population is not managed in specific ACHD centers.^17^ A single-center Australian study evaluated 309 new referrals of patients 16 years or older to a major ACHD center over a 3-year period from 2013 to 2016 and found that 10% of the cohort (65% with moderate/severe CHD) were lost to follow-up and were referred by noncardiac medical professionals.^9^ In addition, in the absence of a structured transition program, those with moderate-severe ACHD under the care of a general cardiologist or primary care physician demonstrated that appropriate management guidelines were not followed compared with a CHD-trained cardiologist (*P* = 0.001) and resulted in significantly increased major/catastrophic adverse outcomes (*P* = 0.002).^9^

Over the period of 1996 to 2000, a total of 22,096 patients with ACHD living in Québec were observed in their utilization of health services.^18^ Ninety-one percent of patients had a primary care physician, and 87% had noncardiology specialist outpatient visits; however, only 55% of the cohort had an outpatient cardiologist appointment with a median of 4 visits over the 5-year period.^18^ This would indicate that patients with ACHD are frequently navigating health care independent of appropriate cardiology oversight.^18^

In the 2004 study of Reid et al.^4^ examining the graduating cohort from the Ontario pediatric reference center, 234 of 360 patients consented to questionnaires about their personal health and habits. Successful transfer occurred in 47% of the cohort. Protective factors against losses to follow-up included patient understanding of the condition and belief that they required specialized follow-up at an ACHD center, comorbid conditions, absence of substance use, dental antibiotic prophylaxis, older age at last pediatric appointment, and attending cardiac appointments without parents or siblings.^4^^,^^19^ There was no difference in the percentage of successful transfer between those who consented to a questionnaire and those who did not.^4^ Whilst the Reid et al. study examined patient-specific factors related to successful transfer of care, our study examined the environment in which the patients live.

In 784 patients with ACHD who underwent CS in Ontario between 2004 and 2015, an assessment of adverse intensive care unit events, 1-year mortality, and socioeconomic factors was examined.^20^ Composite adverse outcomes, defined as renal failure requiring dialysis, prolonged ventilation of over 1 week, and/or in hospital mortality, were more likely to be avoided by those living in the immediate GTA compared with those living outside of the GTA in the rest of Canada (OR: 1.97, *P* = 0.002).^20^ LTFU occurred in 19% of the cohort.^20^ Ontario has universal health care coverage for patient care; however, costs of traveling to the adult referral center and affording lost income while attending appointments are challenging. A recent review of socioeconomic determinants impacting those with ACHD reveals that the financial implications of attending appointments, time off work, travel to access health care, and limitations in insurance are the main determinants of failure of continuity of care.^21^ In addition, those with ACHD were found to have attained a lower level of education and lower level of employment compared with the general population.^21^ These findings are consistent with our postcode-based demographic data collation. Patients navigating cardiac surgical services need essential support services through the perioperative encounter and after discharge. Even with the buffer of remote travel grants, many families cannot afford the delay in reimbursement or have the financial literacy to manage the application process. These grants are restricted and limit the ability to attend clinics or to support a family member at the bedside during a CS admission. Our study period ended 8 months before the declaration of the COVID global pandemic in March of 2020, which imposed economic hardship that most certainly exacerbated barriers to accessing care.

Racial background data are not collected in our hospital information system. There are communities in Ontario that are predominantly First Nations; however, more than 50% of First Nations individuals live in urban settings and 32% live in rural settings.^22^ Individual patients may not use their home Nation’s 6-digit postal address on admission and may be reluctant to self-identify as First Nations for fear of bias. The second character of the postal code is indicative of the coverage of the postal code. A postal code with a "0" in the second character is classified as "rural," and all other postal codes are considered as urban by Canada Post. The last 3 characters together with the forward sortation area identify a specific rural community.^23^ Geographical challenges are not unique to First Nations communities and share the challenge of rural Canada. Although the median distance to the ACHD center was 33.47 km, the range includes patients living in "fly-in" communities over 2000 km away and 12 hours in connecting flights.

A single-center review in Atlanta, Georgia, followed 1514 patients discharged from pediatric care between 2008 and 2010 with a specific referral to transfer care at 1 of 3 specialist centers for ACHD. Overall, only 12.1% transferred care to the referral-affiliated adult hospital although patients with severe complex congenital heart lesion were more likely to successfully transfer (18.7% vs 6.2% for less complex ACHD). Distance to the reference center was a contributing factor.^24^ Distance to the reference center in the United States is comparable to Canada. During our study, access to public transportation in Ontario was curtailed with closure of the passenger rail service Northlander in 2012 and many bus routes in Northern Ontario limiting the public transportation access to the hospital or an airport hub.

Unexpectedly, living in a postal code within the boundaries of Toronto and being employed was common in our failure to transfer study population. Possible explanations include the following: patients are unable to afford the time away from work because of financial or job security reasons, and the proximity of the hospital may be a reassurance, although these are speculative reasons. Over time, being unable to travel to work by car and residing in a lower income neighborhood relate to loss of follow-up.

### Implications of unsuccessful transfer

It is known that loss to follow-up is associated with increased presentations for emergency care and increased mortality. A 2015 review of 7 studies from North America and Europe found that those who had lapses in cardiology follow-up were more likely to have emergency presentations with exacerbation of symptoms and potentially receive new diagnoses.^7^

The impact of living with CHD should not be underestimated. Socioeconomic factors can significantly affect the ability of patients to have a long quality of life. A voluntary online survey of 588 adults in Australia with CHD and 1091 caregivers of children with CHD in 2017-2018 showed that 40% had to travel >200 km to access ACHD treatment.^25^ Of this population with ACHD, 20% had complex lesions, yet only 53% recalled receiving a referral from a pediatric cardiologist to an ACHD cardiologist for their ongoing treatment. The median age of respondents was 39 years. A total of 70% respondents reported being employed; however, 31% reported needing 10 or more days off work within the past year because of their condition. Of all respondents, 30% believed that their ACHD limited work opportunities. Out-of-pocket expenses due to the management of their CHD were estimated at a cost of AUD$2500 (AUD$0-21,000) within the free public access Australian health care system.^25^

A recent comprehensive review of transfer and transition of patients with ACHD from pediatric to adult care highlights patient-specific risk factors but also risk factors related to the patient’s community and resources within the community.^5^

### Limitations

The conclusions of this study should be interpreted considering several limitations. First, the socioeconomic variables used in the analysis are based on postal code–level data derived from Environics Analytics. Although these data provide useful estimates of neighborhood characteristics, they may not accurately reflect individual-level socioeconomic status. Moreover, Environics data, though detailed and commonly used in marketing and policy research, are less frequently applied in health services research compared with standardized indices such as the Canadian Index of Multiple Deprivation or the Ontario Marginalization Index and have not been comprehensively validated in this context.

Second, we were unable to determine outcomes for the 11% who failed to transfer to adult care and the 25% who were lost during follow-up. These knowledge gaps include critical information such as mortality outcomes and potential unmet needs for cardiac surgical services. Although privacy regulations prevented direct patient contact to ascertain these outcomes, future research leveraging provincial health registries would provide valuable insights into these vulnerable patient subgroups and strengthen our understanding of transition barriers.

Lastly, the study period may not reflect current practice patterns or policy changes that have occurred since data collection. Health care delivery, referral pathways, and access to surgical care may have evolved, which could limit the generalizability of our findings to the present-day context.

In addition, neither patient sex nor gender was accounted for in the cohort analysis.

Despite these limitations, we believe that our findings offer important insights into neighborhood-level factors associated with cardiac surgical transfers, and they lay the groundwork for more analyses in future research.

## Conclusions

Young adults with adverse socioeconomic factors face barriers in transferring from children to adult CHD services, and to maintaining care in adulthood.

Further study is needed on the socioeconomic barriers of the entire ACHD population in accessing cardiac care.
